# Mitochondrial Autophagy Involving Renal Injury and Aging Is Modulated by Caloric Intake in Aged Rat Kidneys

**DOI:** 10.1371/journal.pone.0069720

**Published:** 2013-07-22

**Authors:** Jing Cui, Suozhu Shi, Xuefeng Sun, Guangyan Cai, Shaoyuan Cui, Quan Hong, Xiangmei Chen, Xue-Yuan Bai

**Affiliations:** Department of Nephrology, State Key Laboratory of Kidney Diseases, Chinese PLA General Hospital and Medical College, Beijing, China; Imperial College London, Chelsea & Westminster Hospital, United Kingdom

## Abstract

**Background:**

A high-calorie (HC) diet induces renal injury and promotes aging, and calorie restriction (CR) may ameliorate these responses. However, the effects of long-term HC and CR on renal damage and aging have been not fully determined. Autophagy plays a crucial role in removing protein aggregates and damaged organelles to maintain intracellular homeostasis and function. The role of autophagy in HC-induced renal damage is unknown.

**Methods:**

We evaluated the expression of LC3/Atg8 as a marker of the autophagosome; p62/SQSTM1; polyubiquitin aggregates as markers of autophagy flux; Ambra1, PINK1, Parkin and Bnip3 as markers of mitophagy; 8-hydroxydeoxyguanosine (8-OHdG) as a marker of DNA oxidative damage; and p16 as a marker of organ aging by western blot and immunohistochemical staining in the kidneys of 24-month-old Fischer 344 rats. We also observed mitochondrial structure and autolysosomes by transmission electron microscopy.

**Results:**

Expression of the autophagosome formation marker LC3/Atg8 and markers of mitochondrial autophagy (mitophagy) were markedly decreased in the kidneys of the HC group, and markedly increased in CR kidneys. p62/SQSTM1 and polyubiquitin aggregates increased in HC kidneys, and decreased in CR kidneys. Transmission electron microscopy demonstrated that HC kidneys showed severe abnormal mitochondrial morphology with fewer autolysosomes, while CR kidneys exhibited normal mitochondrial morphology with numerous autolysosomes. The level of 8-hydroxydeoxyguanosine was increased in HC kidneys and decreased in CR kidneys. Markers of aging, such as p16 and senescence-associated-galactosidase, were increased significantly in the HC group and decreased significantly in the CR group.

**Conclusion:**

The study firstly suggests that HC diet inhibits renal autophagy and aggravates renal oxidative damage and aging, while CR enhances renal autophagy and ameliorates oxidative damage and aging in the kidneys.

## Introduction

Diet has been long recognized as a modulator of kidney health in both humans and experimental models [1,2]. Calorie restriction (CR) can retard the progression of many age-associated molecular, physiological, and pathological processes which occur in tissues with high oxidative demand, such as kidney [3–5] skeletal muscle [6], heart [7] and brain [8]. Chronic progressive nephropathy was effectively reduced by restricted caloric intake [9]. Life-long CR can prevent glomerular enlargement, foot process widening, PAS-positive volume expansion, podocyte stress, podocyte loss, and glomerulosclerosis [10]. Studies in the kidneys have shown adult-onset CR can reduce effectively the accumulation of mitochondrial enzyme abnormality [11].

In contrast, feeding mice with a high-calorie diet results in age-related obesity, cardiovascular diseases, and other metabolic disorders, and it shortens lifespan [12].

In the past decade, more and more scientists explored the mechanism of the effect of HC and CR on aging [13–15] **.** However, the effects of long-term HC and CR on renal damage and aging have been not fully determined.

Autophagy is an evolutionarily conserved process in eukaryotic organisms. Cytoplasmic constituents are sequestered in double-membrane structures to form autophagosomes, which fuse with lysosomes to form autolysosomes. The cytoplasmic components are degraded by acid hydrolases, and the degradation products are released into the cytosol and recycled into biological structures or to supply energy during periods of starvation [16]. Autophagy is critical for survival during nutrient deprivation, as it enables recycling of macromolecules to provide new nutrients and energy in yeast and mammals. Another key function of autophagy is to remove damaged organelles such as mitochondria and aberrant macromolecules, to prevent further injury to cells. Therefore, impairment of autophagy will lead to a progressive accumulation of damaged macromolecules and organelles in somatic cells, increased oxidative damage and accelerated aging [17].

Recent studies have revealed that both chronic (∼ 4 to 5 months) and short-term (4 or 8 weeks) high-fat diets can inhibit autophagy in the hypothalamus, skeletal and cardiac muscle. Other studies have demonstrated that 8–20 weeks of a high-fat diet can induce autophagy in pancreatic β-cells [18–21]. The effect of a HC diet on autophagy in other organs, including the kidneys, has not been determined. It is also not clear whether autophagy, especially mitochondrial autophagy (mitophagy), is involved in renal organ injury and aging modulated by a long-term HC diet. We speculate that a HC diet might aggravate damage and aging via inhibition of autophagy in aged kidneys. In contrast, CR might ameliorate oxidative damage and aging via restoration of autophagy in aged kidneys.

In the present study, for the first time, we investigated the effect of a long-term (20-month) HC diet or CR on mitochondrial ultrastructure and the expression of LC3/Atg8 (as a marker of the autophagosome); p62/SQSTM1 and polyubiquitin aggregates (as markers of autophagy flux); Ambra1, PINK1, Parkin and Bnip3 (as markers of mitophagy); 8-hydroxydeoxyguanosine (8-OHdG) (as a marker of DNA oxidative damage); and p16 (as a marker of organ aging) in the kidneys of 24-month-old male Fischer 344 rats.

## Materials and Methods

### Animals

Male 3-month-old Fischer 344 rats were purchased. After adaptive feeding for one month in Experimental Animal Center of Chinese PLA General Hospital, these rats were randomly divided into three groups, and respectively given different diet interventions for 20 months. Control rats (n  = 20) were fed a normal rat chow of 3.42 kcal/g (Keao, Beijing, China). HC rats (n  = 25) were fed a modified chow of 4.51 kcal/g (Keao, Beijing, China). CR rats (n  = 16) were fed 70% of the calorie intake of control rats. All animals were maintained in individual plastic cages and had free access to water. All experiments involving animals were approved by the Institutional Animal Care and Use Committee at the Chinese PLA General Hospital. Urine was collected in a tube using metabolic cages over a 24-h period and stored at −80°C.

At 24 months of age, six rats per group were anesthetized via intraperitoneal injection of sodium pentobarbital (40 mg/kg). The abdomen was opened and blood was collected from the abdominal aorta. Kidney tissues were removed and perfused with ice-cold, isotonic phosphate-buffered saline (PBS; pH 7.4) to remove any remaining blood. A portion of the renal tissue was fixed in 2.5% glutaraldehyde for transmission electron microscopy. The remaining tissues were immediately frozen in liquid nitrogen and stored at –80°C until further processing. The numbers of CON, CR and HC rats at the age of 3-month and 24-month and the mortality during the experiment were shown in table 1. We randomly sacrificed six rats in each group when the rats were 24-month-old. The rest rats in each group were continued feeding and used in other subsequent experiments. Metabolic parameters and renal function in table 2 were done and presented in only the rats sacrificed.

**Table 1:** The numbers of CON, CR and HC rats at the age of 3-month and 24-month.

Group	3-month-old		24-month-old		Mortality during the 20 month
CON		20		14	6
CR		16		14	2
HC		25		8	17

CON, control animals; CR, calorie-restricted diet; HC, high-calorie diet.

**Table 2:** Effect of diet on metabolic parameters and renal function in the aged rats.

	CON	CR	HC
BMI (kg/m2)	9.18±0.74	7.60±0.33	7.50±1.95
Kidney weight (g)	2.73±0.18	2.95±0.37	3.70±0.18*
Serum glucose (mmol/L)	6.63±1.924	6.31±0.92	5.24±1.56
Serum creatinine (µmol/L)	33.12±3.63	27.67±3.12	42.8±11.0*
Serum triglyceride (mmol/L)	2.56±0.85	0.53±0.18*	3.56±1.36
Serum cholesterol (mmol/L)	4.42±0.42	2.74±0.49*	18.57±4.35*
Urine protein/creatinine ratio (mg/mmol)	529.35±139.9	256.37±98.38*	1178.53±149.34*

BMI, body mass index; CON, control animals; CR, calorie-restricted diet; HC, high-calorie diet. Data are presented as means ± SD (n  = 6), *P<0.05 *vs.* CON.

### Periodic Acid-Schiff (PAS) Staining

Kidneys from the rats were excised, fixed in 4% paraformaldehyde, embedded in paraffin and sectioned at 4 µm for histological staining with periodic acid-Schiff followed by examination under the microscope.

### Western Blot

The tissues from the cortex of the kidneys were lysed in RIPA buffer (50 mM Tris-Cl [pH 7.6], 150 mM NaCl, 1% NP-40, 0.1% SDS, 0.5% deoxycholic acid, 1 µg/mL leupeptin, 1 µg/mL aprotinin, and 0.5 mM phenylmethylsulfonyl fluoride) for 30 min on ice prior to centrifugation at 12,000 rpm for 30 min at 4°C. The protein concentrations were determined using a BCA protein assay kit (Thermo Fisher Scientific, Rockford, IL, USA). Proteins were separated by sodium dodecylsulfate–polyacrylamide gel electrophoresis using a 6–15% acrylamide resolving gel and transferred to nitrocellulose membranes. Membranes were blocked in TBS-T (0.1% Tween-20) containing 5% milk for 1 h at room temperature followed by incubation with primary antibody at 4°C overnight. A rabbit anti-LC3 polyclonal antibody (1∶2000; Sigma, St. Louis, MO, USA), mouse monoclonal anti-p16 (1∶200; Santa Cruz Biotechnology, Santa Cruz, CA, USA), mouse monoclonal anti-ubiquitin (1∶2000; MABtech, Nacka Strand, Sweden), rabbit polyclonal anti-Parkin (1∶200; Santa Cruz Biotechnology, Santa Cruz, CA, USA), rabbit polyclonal anti-PINK1 (1∶200; Santa Cruz Biotechnology, Santa Cruz, CA, USA) and mouse monoclonal anti-p62 (1∶200; Santa Cruz Biotechnology, Santa Cruz, CA, USA), rabbit polyclonal anti-Bnip3 (1∶5000; Abcam, Cambridge, MA, USA), rabbit polyclonal anti-Ambra1 (1∶5000; Abcam, Cambridge, MA, USA), or β-actin antibody (1∶5000; Sigma, St. Louis, MO, USA) was used to detect the corresponding proteins. Blots were subsequently probed with horseradish peroxidase-conjugated anti-mouse or anti-rabbit IgG (Beyotime Institution of Biotechnology, Beijing, China) at 1∶1000–5000 dilutions. Immunoreactive bands were visualized by enhanced chemiluminescence and densitometry was performed using the Quantity One software (Bio-Rad Laboratories, Hercules, CA, USA).

### Immunohistochemistry

The kidneys were fixed in 10% formaldehyde overnight at 4°C and processed for paraffin-embedding following standard procedures. Sections were cut at 3-µm thicknesses. For immunohistochemical analysis, some tissue sections were subjected to antigen retrieval by microwaving or autoclaving for 10 or 15 min in 10 mM sodium citrate buffer [pH 6.0]. Endogenous peroxidase activity was blocked by incubation with 3% hydrogen peroxide for 10 min. After PBS washing, sections were incubated with 1.5% normal goat serum for 20 min, followed by incubation with mouse monoclonal anti-8-OHdG antibody (1∶50; Santa Cruz Biotechnology Inc., CA, USA) and rabbit polyclonal anti-LC3 (1∶50; Sigma, St. Louis, MO, USA) overnight at 4°C. After three washes with PBS, the samples were incubated with biotin-conjugated goat anti-mouse IgG (Invitrogen Corporation, CA, USA) for 30 min at room temperature. After washing in PBS, the sections were incubated with streptavidin-conjugated peroxidase (Invitrogen Corporation, CA, USA) 30 min at room temperature. After PBS washing, the sections were incubated with DAB (Invitrogen Corporation, CA, USA) followed by examination under the microscope.

### Senescence-associated β-galactosidase (SA-β-gal) Staining

Four-micrometer kidney sections were fixed in 2% formaldehyde/0.2% glutaraldehyde at room temperature for 15 min. The sections were washed twice in PBS and incubated in freshly prepared SA-β-gal staining solution (1 mg/ml X-gal, 40 mM citric acid/sodium phosphate [pH 6.0], 5 mM potassium ferrocyanide, 5 mM potassium ferricyanide, 150 mM NaCl, and 2 mM MgCl_2_) overnight at 37°C without CO_2_. Tissue sections were counterstained with eosin and examined under the light microscopy.

### Transmission Electron Microscopy

Kidneys were cut into tissue blocks (1 mm^3^) and fixed in 2.5% glutaraldehyde in 0.01 mol/L phosphate buffer at 4°C, followed by 2% osmium tetroxide. They were then dehydrated in a series of graded ethanol solutions. Ethanol was then substituted with propylene oxide and the tissue was embedded in epoxy resin. Ultrathin sections were double-stained with uranyl acetate and lead and examined under a JEM1200EX transmission electron microscope (JOEL) at 80 kV.

### Statistical Analysis

Analyses of all data were performed using the SPSS ver. 13.0 (SPSS, Chicago, IL, USA) software. Data are expressed as means ± standard deviation (SD). Comparisons among groups were made using analysis of variance. Values of *P*<0.05 were considered to indicate statistical significance.

## Results

### Effects of Diet on Kidney Function in the Aged Rats

We analyzed changes in the metabolic parameters and renal functions in the control, HC and CR rats (**Table 2**). The level of serum glucose and body mass index (BMI) were unchanged among the three groups. Kidney weight, serum creatinine, cholesterol and the urine protein/creatinine ratio were increased significantly in the HC rats. The serum levels of triglyceride and cholesterol, and the urine protein/creatinine ratio were significantly decreased in the CR group (**Table 2**).

### Periodic Acid-Schiff (PAS) Staining Results

**Figure 1:**
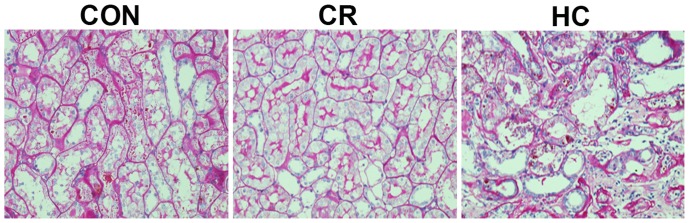
PAS staining of the renal tissues from the CON, CR and HC Fischer 344 rats. CON, control animals; CR, calorie-restricted diet; HC, high-calorie diet. Magnification, ×400.

Periodic acid-Schiff (PAS) staining results showed that there were more obvious tubulointerstitial damages as well as a moderate inflammatory response in HC group compared to the CON group (**Figure 1**). In CR group, the pathological changes were alleviated.

### Effects of Diet on Autophagy Activity in Aged Rat Kidneys

#### The autophagosome formation marker LC3/Atg8

**Figure 2:**
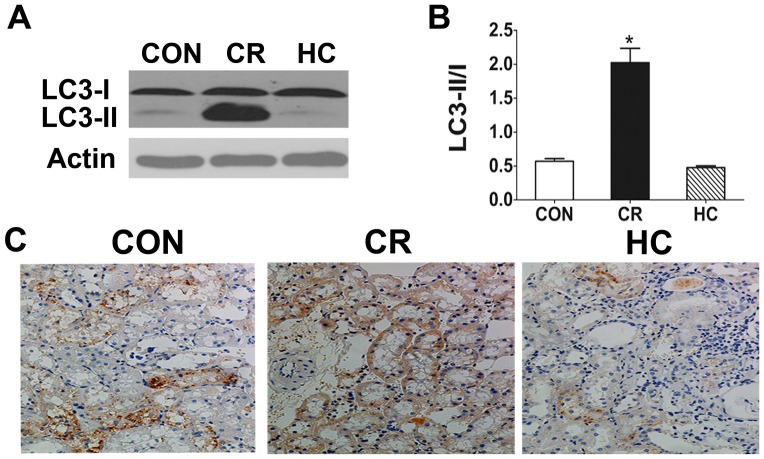
LC3 was decreased in HC kidneys and increased in CR kidneys compared with CON kidneys. LC3 expression was analysed by Western blotting in the renal tissues of the CON, CR and HC Fischer 344 rats. **a:** Theexpression of LC3-I and LC3-II proteins was detected by Western blot analysis.**b:** The ratio of the LC3-II to LC3-I bands was analyzed. The protein expression data are presented as the mean ± SD (n  = 6). *p<0.05 vs. CON.**c:** Immunohistochemistry staining results for LC3 proteins and 8-OHdG in the kidneys of the CON, CR and HC Fischer 344 rats were scanned by a microscope.CON, control animals; CR, calorie-restricted diet; HC, high-calorie diet.

The microtubule-associated protein 1 light chain 3 (LC3) is required for the formation of autophagosomes, especially the expansion of early autophagosomes [22]. The cytosolic isoform of LC3 (LC3-I) is conjugated to phosphatidylethanolamine through two consecutive ubiquitination-like reactions that are catalyzed by the E1-like enzyme Atg7 and the E2-like enzyme Atg3 to form LC3-II [23]. LC3-II is the only protein marker that is reliably associated with completed autophagosomes, but it is also localized to phagophores. Thus, LC3-II formation is recognized as a marker of autophagosomes in cell or animal experiments [24,25]. LC3-I and -II are general markers of autophagic membranes and te LC3II/I ratio is correlated with autophagic flux [26]. We first observed the expression of LC3 by immunohistochemistry and western blot. Western blot analysis revealed that LC3-II/I expression was increased significantly in CR kidneys (**Figure 2**). The results showed that expression of LC3 was decreased moderately in HC kidneys and increased in CR kidneys compared with control (CON) kidneys (**Figure 2**). These results indicate that autophagy increases in the kidneys of the CR rats.

#### Autophagy degradation markers, p62/SQSTM1 and polyubiquitin aggregates

**Figure 3:**
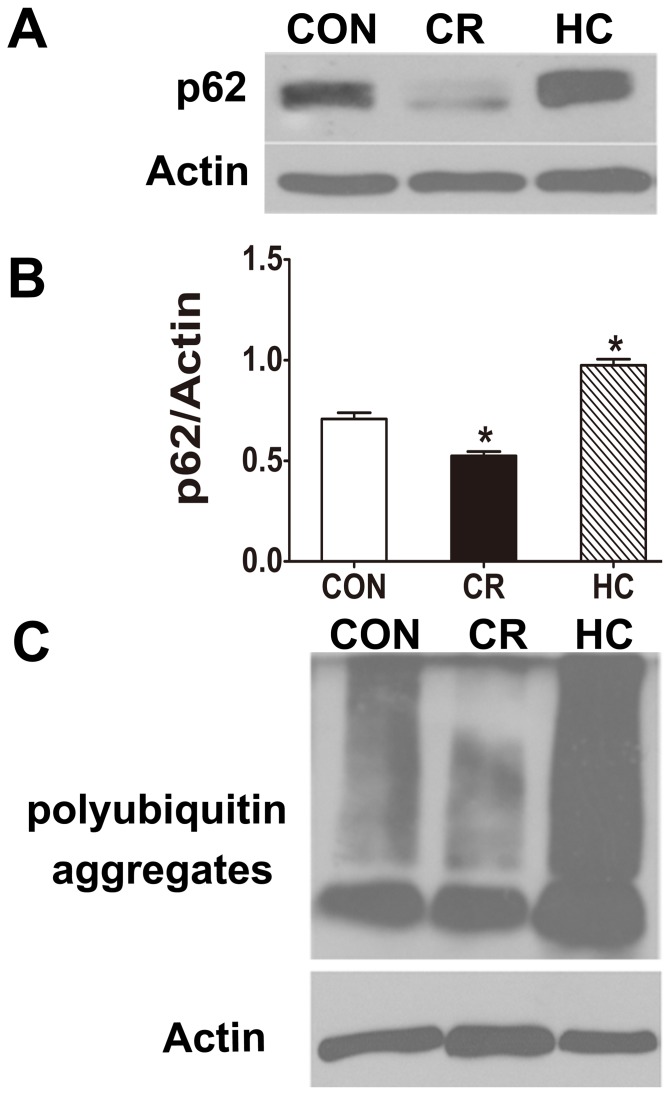
P62 was decreased in HC kidneys and increased in CR kidneys compared with CON kidneys. The expression of p62/SQSTM1 and polyubiquitin aggregates was analyzed by Western blotting in kidneys from CON, CR, and HC Fischer 344 rats. **a:** The expression of p62/SQSTM1 was detected by Western blotting. **b:** Quantitative analysis of the band density for p62/SQSTM1.**c:** The expression of polyubiquitin aggregates (poly-UB) was analyzed by Western blotting. The protein expression data are presented as the mean ± SD (n  = 6). *p<0.05 vs. CON. CON, control animals; CR, calorie-restricted diet; HC, high-calorie diet.

Autophagic flux refers to the complete process of autophagy including formation of autophagosomes, fusion of autophagosomes with lysosomes and their subsequent breakdown [27]. Autophagic flux can be measured by determining the declining abundance of autophagic substrates such as p62/sequestosome 1 (SQSTM1), which is sequestered in autophagosomes upon its direct interaction with LC3. P62/SQSTM1, a ubiquitin-binding protein, interacts with LC3 [28] and mediates the degradation of polyubiquitinated protein aggregate by the autophagy-lysosome pathway in mammalian cells. Therefore, p62 and polyubiquitinated protein aggregates may act as markers of autophagic degradation. The increases in p62 and polyubiquitinated protein aggregates in the cells represent a decrease in autophagy activity. Our results showed that the expression of p62/SQSTM1 and polyubiquitin aggregates were increased significantly in HC rat kidneys, whereas both were decreased significantly in CR rat kidneys (**Figure 3**), indicating that autophagy function decreases in HC and that CR can enhance autophagy in aged rat kidneys.

### Effects of Diet on Mitophagy Function in Aged Rat Kidneys

#### Parkin and PINK1

**Figure 4:**
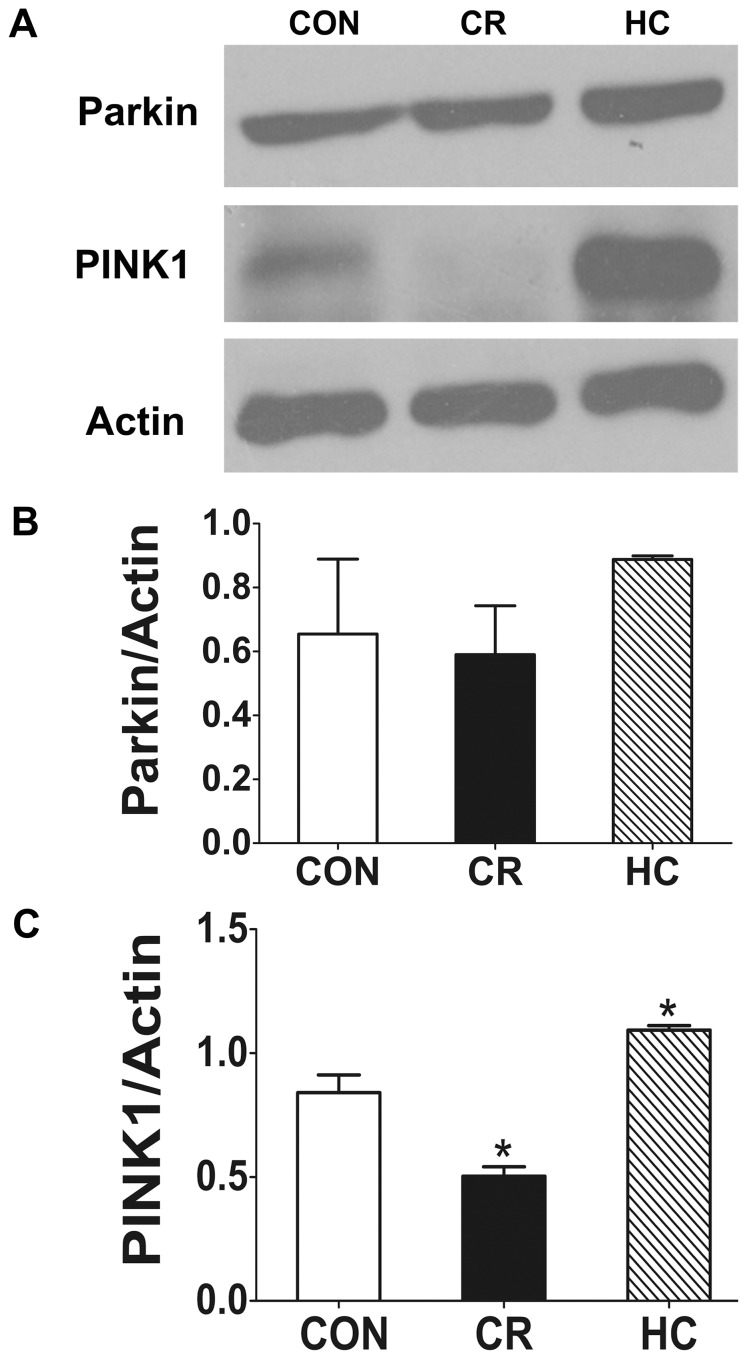
PINK1 was decreased in HC kidneys and increased in CR kidneys compared with CON kidneys. The expression of Parkin and PINK1 was analyzed by Western blotting in the kidneys of CON, CR, and HC Fischer 344 rats. **a:** The expression of Parkin and PINK1 was detected by Western blotting. **b:**Quantitative analysis of the band density for Parkin. **c:**Quantitative analysis of the band density for PINK1. The protein expression data are presented as the mean ± SD (n  = 6). *p<0.05 vs. CON. CON, control animals; CR, calorie-restricted diet; HC, high-calorie diet.

PTEN-induced kinase 1 (PINK1) and Parkin are mutated in many cases of early onset familial Parkinson’s disease (PD). Recent work suggests that PINK1 and Parkin function in the same pathway to maintain mitochondrial integrity [29–31]. Genetic approaches have revealed that PINK1 functions upstream of Parkin in the regulation of mitochondrial dynamics [32]. PINK1, as a sensor of mitochondrial damage and initiator of mitophagy, translocates from the cytosol to the depolarized mitochondria and subsequently triggers mitochondrial elimination through mitophagy. Our results show that the expression of PINK1 was increased significantly in HC kidneys, indicating that mitochondrial damage is increased. In contrast the expression of PINK1 was decreased significantly in CR kidneys (**Figure 4**), indicating that CR can mitigate the mitochondrial damage. However, the expression of Parkin among the CON, CR and HC groups was not significantly different (**Figure 4**).

#### Bnip3

**Figure 5:**
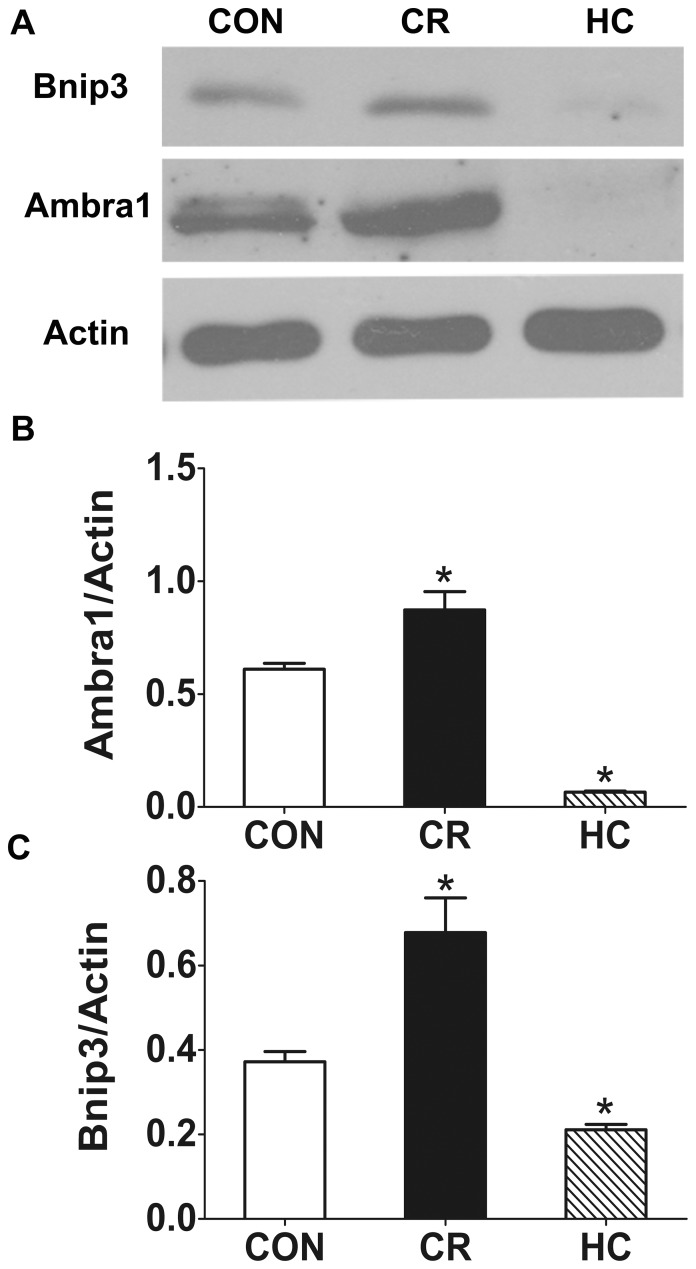
Bnip3, Ambra1 decreased in HC kidneys and increased in CR kidneys compared with CON kidneys. The expression of Bnip3 and Ambra1was analyzed by Western blotting in the kidneys of CON, CR, and HC Fischer 344 rats. **a:** The expression of Bnip3 and Ambra1 was detected by Western blotting. **b:**Quantitative analysis of the band density for Bnip3. **c:**Quantitative analysis of the band density for Ambra1. The protein expression data are presented as the mean ± SD (n  = 6). *p<0.05 vs. CON.CON, control animals; CR, calorie-restricted diet; HC, high-calorie diet.

Bnip3 is anchored in the outer mitochondrial membrane via its C-terminal transmembrane domain (TMD), while the N-terminus faces the cytosol. The C-terminal TMD is essential for targeting Bnip3 to the mitochondria, homodimerization and pro-apoptotic activity. Interestingly, the N-terminus of Bnip3 contains a WXXL-like motif that might be important in binding to Atg8-family proteins [33,34]. Binding of Bnip3 to Atg8 proteins such as LC3 might serve to dock mitochondria to the autophagosomes, thereby ensuring their removal. Our results show that the expression of Bnip3 was decreased significantly in HC kidneys (**Figure 5**), indicating that HC may inhibit mitophagy. In contrast, the expression of Bnip3 was increased significantly in CR rat kidneys (**Figure 5**), indicating that CR may enhance mitophagy.

#### Ambra1

Ambra1 (activating molecule in Beclin 1-regulated autophagy) interacts with Parkin, a protein that promotes autophagy in the vertebrate central nervous system. Ambra1 is recruited in a Parkin-dependent manner to perinuclear clusters of depolarized mitochondria and contributes to their selective autophagic clearance [35]. The only autophagy-regulating protein among the identified Parkin interactors was Ambra1. Our results showed that the expression of Ambra1 was significantly decreased in HC rat kidneys, indicating that HC may inhibit mitophagy. In contrast, the expression of Ambra1 was significantly increased in CR rat kidneys (**Figure 5**), indicating that CR may enhance mitophagy function.

### Effects of Diet on Mitochondrial Oxidative Damage in the Kidneys

**Figure 6:**
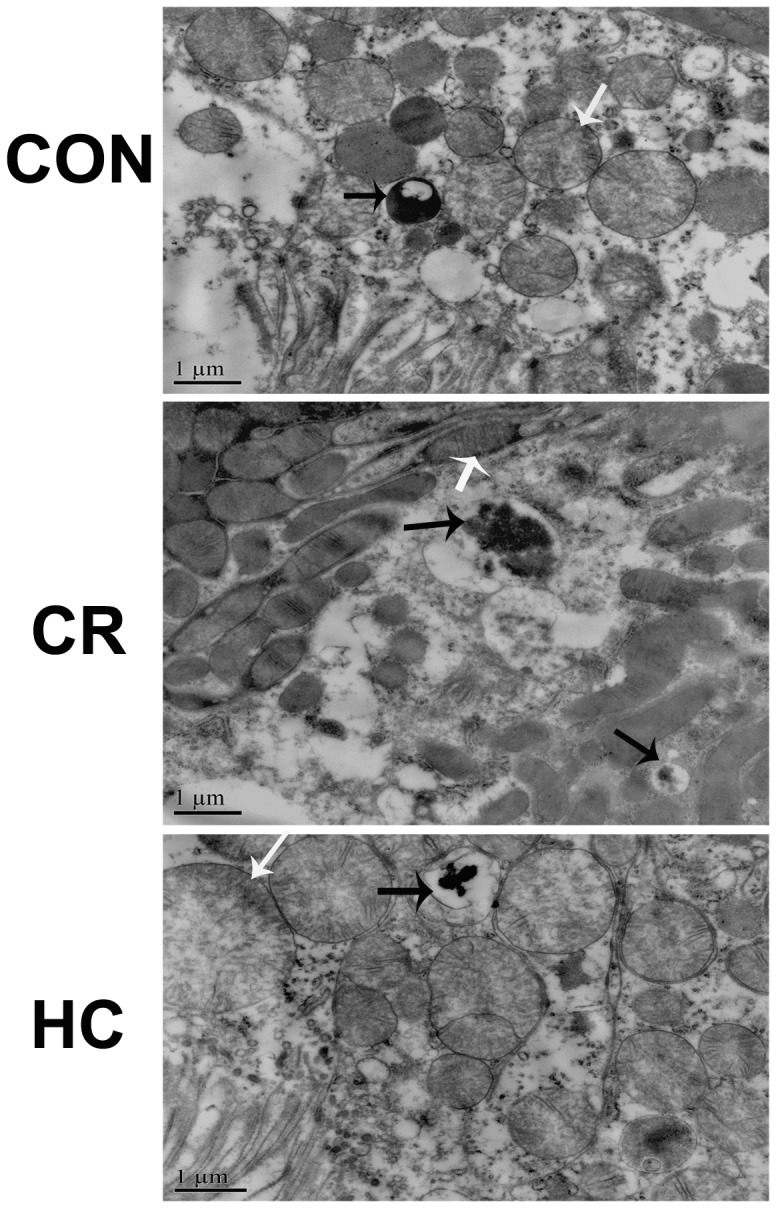
The damage of mitochondrial structures was increased in HC kidneys and decreased in CR kidneys. Analysis of mitochondrial structures and autolysosomes by transmission electron microscopy (TM) in the renal tissues of CON, CR, and HC Fischer 344 rats. White arrows indicate damagedmitochondria; black arrows indicate autolysosomes.CON, control animals; CR, calorie-restricted diet; HC, high-calorie diet.

**Figure 7:**
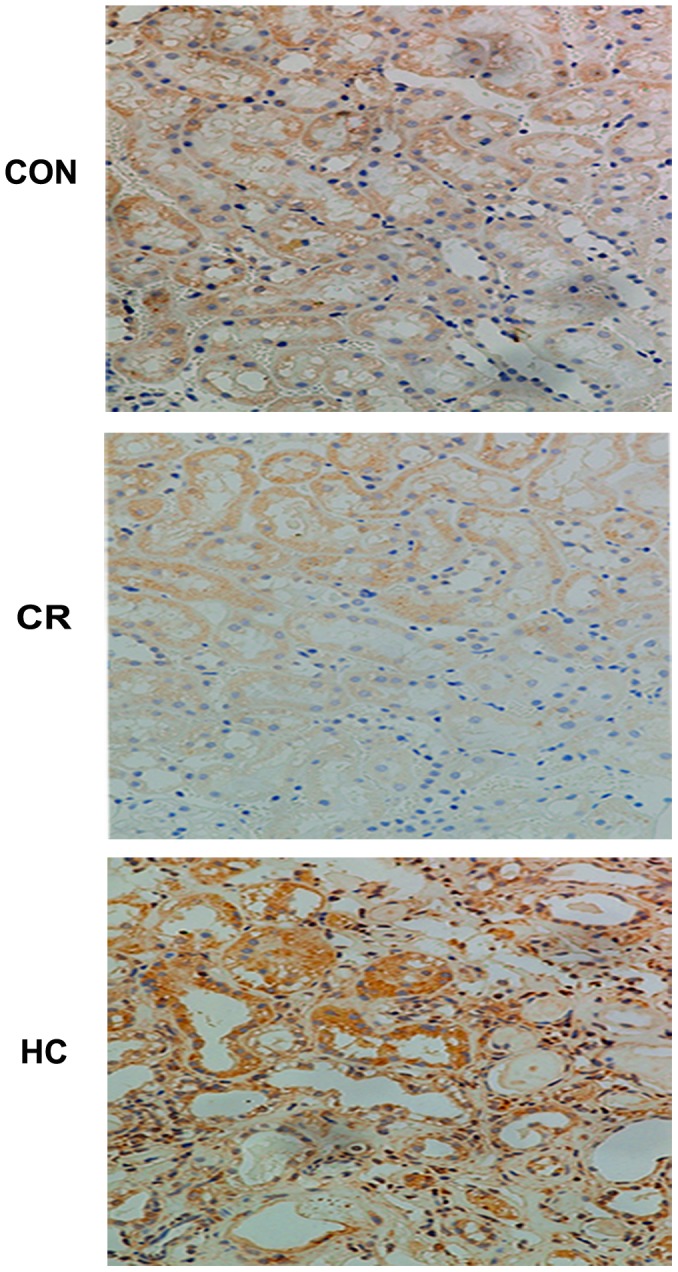
The level of 8-OHdG was increased in HC kidneys and decreased in CR kidneys. Immunohistochemistry staining results for LC3 proteins and 8-OHdG in the kidneys of the CON, CR and HC Fischer 344 rats were scanned by a microscope. CON, control animals; CR, calorie-restricted diet; HC, high-calorie diet.

Oxidative stress, including reactive oxygen species (ROS), can damage both cellular macromolecules such as DNA, proteins, and lipids, and mitochondrial structure. In this study, we further observed the effect of diet on mitochondrial structures and the number of autolysosomes using transmission electron microscopy. The results showed obvious injury to mitochondrial structures, such as swelling and disintegration of cristae in the 24-month-old CON groups. There was also more severe mitochondrial damage and fewer autolysosomes in 24-month-old HC rats, whereas the 24-month-old CR groups exhibited relatively mild damage and more autolysosomes (**Figure 6**). We further evaluated the effects of diet on mitochondrial DNA in renal tissues by analyzing the level of 8-OHdG, a sensitive biomarker of oxidative DNA damage, especially mitochondrial DNA (mtDNA) [36]. We found that the 8-OHdG level was increased in the kidneys of HC rats, but decreased in the CR group (**Figure 7**).

### Effects of Diet on Senescence Markers in the Kidneys of Aged Rats

**Figure 8:**
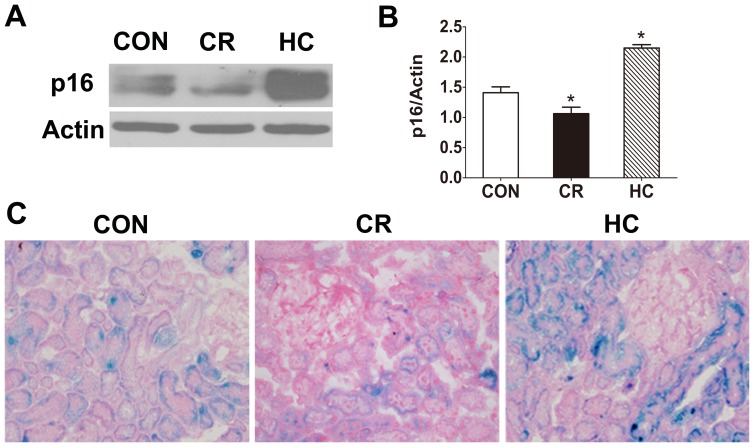
The levels of p16 and SA-β-gal increased in HC kidneys and decreased in CR kidneys. The expression of the senescence biomarkers p16 and senescence-associated-galactosidase wasanalyzed in the kidneys of CON, CR, and HC Fischer 344 rats. **a:** Western blot results for p16 protein. **b:** Quantitative analysis of the band density for p16. The protein expression data are presented as the mean ± SD (n  = 6). *p<0.05 vs. CON. **c:**Senescence-associated-galactosidase staining (magnification ×400). Blue precipitation in the cytoplasm was observed in the senescent cells.CON, control animals; CR, calorie-restricted diet; HC, high-calorie diet.

p16 is a robust biomarker and a possible effector in mammalian renal aging [37]. The expression of p16 was measured in aged kidneys from the three groups of rats. The expression of p16 increased significantly in HC kidney tissue compared to CON tissue (**Figure 8**), and decreased significantly in CR kidney tissue (**Figure 8**) compared to CON tissue. We also determined the expression of another well-defined *in vivo* marker of senescence, SA-β-gal, which results in detectable galactosidase activity in senescent cells and tissues [38,39]. SA-Beta-Gal activity predominantly increased in the cortical tubules in response to diabetes. But, SA-Gal activity was not evenly distributed throughout the cortex. Some areas were more prominent than others. SA-Beta-gal staining appears as bright-blue granular staining into the cytoplasm of tubular epithelial cells, mainly proximal tubules [40,41]. As shown in **Figure 8**, positive SA-β-gal staining was markedly elevated in the kidneys of HC rats and decreased in the kidneys of CR rats, compared with CON control rats.

## Discussion

Previous study has shown that tubulointerstitial cell proliferation and interstitial accumulation of various extracellular matrix proteins progressively increases with increasing age in Milan rats [42]. In recent years, a close relationship between metabolic dysfunction, oxidative damage and aging of tissues and organs has been established. Adult-onset calorie restriction (AL-CR) would reduce or delay mtDNA mutations and the electron transport system enzyme abnormalities accumulation, and slow these age-related changes, such as glomerulosclerosis and tubularatrophy compared with ad libitum-fed rat [11]. In other study, it has been found that calorie restriction can alleviate the glomerular enlargement and glomerulosclerosis in the aging rat [10]. However, the effects of long-term high calorie diet on renal damage and aging have been not determined.

Compared with the above studies, in the present study, we mainly observed the effects of long-term diet (from 3-month-old to 24-month-old) intervention with either HC diet or CR on autophagy (especially mitophagy), oxidative damage and aging in the kidneys (mainly kidney tubular epithelial cells) of aged rats (24-month-old). We found that long-term HC markedly inhibited renal autophagy function, and that CR intervention may significantly increase the level of autophagy in the aged rat kidneys.

Eukaryotic cells have two major degradation systems, the autophagy–lysosome and proteasome pathways. Proteasomal degradation has high selectivity; the proteasome generally recognizes only ubiquitinated substrates, which are primarily short-lived proteins [43]. The autophagy-lysosomal pathway is responsible for the degradation of long-lived proteins and for the elimination of damaged or functionally redundant organelles [44]. Autophagy plays a role in multiple physiological mechanisms, including starvation, clearance of aggregated or mis-folded proteins, cell growth, anti-aging, and innate immunity. Thus, deregulation of autophagy is involved in many diseases including cancer, cardiovascular, muscular, and neurodegenerative disorders [45,46]. In recent years, many studies focus on the relationship between autophagy and dysfunction and aging in kidneys. For example, the role of autophagy in maintaining podocyte homeostasis has been shown in the aging Atg5^−/−Δpodocyte^ mice (20- to 24-month) [47]. In addition, it has been recently shown that adult-onset calorie restriction (at the age of 12-month-old rats) attenuates mitochondrial oxidative damage in aged kidney, enhances autophagy and enhances hypoxia-induced expression of BCL2/adenovirus E1B 19-kDa interacting protein 3 in aged kidney [48].

In multicellular organisms, an important function of autophagy is the clearance of damaged or aged proteins or organelles, such as mitochondria. Recent studies suggest that this degradative process is selective and mediated by the mammalian protein p62/sequestosome 1 (SQSTM1) [46]. p62 directly binds both poly- or mono-ubiquitin and LC3, and links the ubiquitinated substrates to the autophagy machinery for autophagic degradation. The measurement of autophagic substrates (p62 and polyubiquitinated protein aggregates) has been used to evaluate autophagic flux [49]. A previous experiment demonstrated that when autophagy function is impaired, the level of p62 is increased [50]. Therefore, p62/SQSTM1 and polyubiquitinated protein aggregates may be seen as markers of autophagy function. Inhibition of autophagy correlates with increased levels of p62 in mammals and *Drosophila*, suggesting that the steady-state level of this protein reflects the autophagic status [51]. Similarly, decreased p62 levels are associated with autophagy activation. In this study, we found that the level of p62/SQSTM1 and polyubiquitinated protein aggregates increased in the HC rat kidneys and decreased in the CR rat kidneys, indicating that HC may inhibit autophagy and that CR may elevate autophagic activity in the kidneys.

Mitochondrial autophagy (mitophagy) is the only intracellular degradative mechanism for the removal of damaged mitochondria. Mitophagy was proposed to decrease the potential oxidative damage from defective mitochondria [52]. PINK1 and Parkin are mediators of the selective degradation of dysfunctional mitochondria by mitophagy. Parkin (E3 ubiquitin ligase) has been found to selectively bind only to damaged mitochondria and to mediate a mitophagy quality control pathway. Previous results showed that mitochondrial contents of both full-length 63-kDa and cleaved 52-kDa PINK1 species increased after dissipation of ΔΨm, suggesting that mitochondrial depolarization enhances PINK1 stability [53]. Thus PINK1 may play a vital role in maintaining mitochondrial homeostasis by operating as a sensor of mitochondrial damage and initiator of mitophagy [33]. The increased level of PINK1 in the HC group may indicate increased mitochondrial damage.

Bnip3 promotes translocation of Parkin to the damaged mitochondria from the cytoplasm [54], and Ambra1 is the only autophagy-regulating protein among the identified Parkin interactors. Our results indicate that HC could significantly decrease the expression of Bnip3 and Ambra1, and CR intervention could increase Bnip3 expression. At present, the relationship between Bnip3 and kidney aging is not clear. In 2010, one study found that adult-onset and long-term CR in mice attenuated hypoxia-associated mitochondrial and renal damage by enhancing Bnip3-dependent autophagy. Bnip3 expression was significantly enhanced in renal proximal tubular cells of CR mice [48], which was consistent with our results. These above results indicated that HC inhibits autophagy, especially mitophagy, and that CR intervention can increase mitophagy in the aged kidney.

Mitochondria are a major intracellular source of reactive oxygen species (ROS), and mitophagy is the only intracellular degradative mechanism for the removal of damaged mitochondria. Therefore, if mitophagy function is impaired and damaged mitochondria are not removed promptly, they will produce more ROS and further aggravate injury and aging of tissues and organs. In this study, we found that an HC diet results in more severe mitochondrial damage and increased levels of 8-OHdG and p16, while CR may ameliorate the mitochondrial damage and decrease the levels of 8-OHdG and p16 in the kidneys. This suggested that the excess energy intake from a HC diet might cause oxidative damage and aging via inhibition of mitochondrial autophagy in the kidneys, whereas restriction of caloric intake could increase mitochondrial autophagy and mitigate oxidative damage and aging in the kidneys.

Previous animal studies have shown that endoplasmic reticulum (ER) stress or oxidative stress induces early phase adaptive autophagy upregulation, which helps maintain intracellular homeostasis by disposing of a number of harmful molecules, such as cytosolic proteins damaged by ROS, and mitochondria [55]. However, when intracellular stresses remain unresolved, prolonged autophagy upregulation progresses into an autophagy defect [56]. In our studies, we found that autophagy function was decreased in aged kidneys after consumption of a long-term HC diet. However, the exact mechanisms by which an HC diet induces oxidative injury of tissues and organs remain to be elucidated. The role of autophagy in renal injury induced by a HC diet is not clear. In the future experiment, we will further define the causality between calorie restriction or high-calorie diet and autophagy, mitophagy and oxidative damage by conducting diet intervention against autophagy-related genes deficient mice.

Studies in recent years have showed that deficiency and dysfunction in autophagy are implicated in the pathogenesis and progression of some renal diseases, such as glomerulosclerosis, renal ischemia/reperfusion injury, cyclosporine nephrotoxicity, and cisplatin nephrotoxicity [47,57,58]. Improved autophagy may decrease renal damage. Our results are helpful in understanding the role of autophagy in the onset and development of kidney diseases and aging and in exploring new therapeutic methods for metabolic syndrome associated with chronic kidney disease.
